# User Preferences and Persona Design for an mHealth Intervention to Support Adherence to Cardiovascular Disease Medication in Singapore: A Multi-Method Study

**DOI:** 10.2196/10465

**Published:** 2019-05-28

**Authors:** Victoria Haldane, Joel Jun Kai Koh, Aastha Srivastava, Krichelle Wei Qi Teo, Yao Guo Tan, Rui Xiang Cheng, Yi Cheng Yap, Pei-Shi Ong, Rob M Van Dam, Jie Min Foo, Falk Müller-Riemenschneider, Gerald Choon-Huat Koh, Pin Sym Foong, Pablo Perel, Helena Legido-Quigley

**Affiliations:** 1 Institute of Health Policy, Management and Evaluation University of Toronto Toronto, ON Canada; 2 Saw Swee Hock School of Public Health National University of Singapore Singapore Singapore; 3 Department of Pharmacy National University of Singapore Singapore Singapore; 4 London School of Hygiene and Tropical Medicine London United Kingdom; 5 World Heart Federation Geneva Switzerland

**Keywords:** personas, biopsychosocial personas, qualitative, ASCVD, adherence, patient perspectives

## Abstract

**Background:**

The use of mobile health (mHealth) has gained popularity globally, including for its use in a variety of health interventions, particularly through short message service (SMS) text messaging. However, there are challenges to the use of mHealth, particularly among older users who have a large heterogeneity in usability and accessibility barriers when using technology.

**Objective:**

In order to better understand and conceptualize the diversity of users and give insight into their particular needs, we turned to persona creation. Personas are user archetypes created through data generated from multi-method inquiry with actual target users. Personas are an appropriate yet largely underutilized component of current mHealth research.

**Methods:**

Leveraging data from a multi-method study conducted in Singapore with an ethnically diverse population including Chinese, Malay, and Indian participants, we used a proforma to analyze data from the qualitative component (ie, 20 in-depth interviews) and quantitative component (ie, 100 interviewer-guided surveys). We then identified key characteristics, including technology use and preferences as well as adherence factors, to synthesize five personas reflective of persons over the age of 40 years in Singapore with atherosclerotic cardiovascular disease (ASCVD) or ASCVD risk factors, such as hypertension.

**Results:**

We present five personas typologized as (1) The Quiet Analog, (2) The Busy Grandparent, (3) The Socializer, (4) The Newly Diagnosed, and (5) The Hard-to-Reach. We report on four key characteristics: health care access, medication adherence, mobile phone technology usage (ie, ownership, access, and utilization), and interest in mHealth. Finally, we provide insights into how these personas may be used in the design and implementation of an mHealth intervention. Our work demonstrates how multi-method data can create biopsychosocial personas that can be used to explore and address the diversity in behaviors, preferences, and needs in user groups.

**Conclusions:**

With wider adoption of mHealth, it is important that we consider user-centered design techniques and design thinking in order to create meaningful, patient-centered interventions for adherence to medications. Future research in this area should include greater exploration of how these five personas can be used to better understand how and when is best to deliver mHealth interventions in Singapore and beyond.

## Introduction

The use of mobile phones for health-related purposes (ie, mobile health [mHealth]) has gained popularity globally, and mobile phones are becoming widely used in a variety of health interventions [1]. In particular, short message service (SMS)-based interventions have shown promise due to affordability and wide outreach and have, therefore, been applied in health promotion and medication adherence [2-6]. However, there are challenges to the use of mHealth, particularly among older adult users who may face a wide variety of perceptual and impairment barriers to access, as well as technology usability challenges [7,8]. Efforts to address these challenges are complicated by differing degrees of effect on each user, as older adults are highly heterogenous in their experience and ability to use technology [9]. In light of this, there is a need for user-centered design in mHealth interventions in order to bridge the digital divide and enable appropriate and effective interventions that reach users who need them most [10].

Personas are a key feature of user-centered design and are used widely in Web design fields, including product evaluation, design, usability testing, and marketing and product development, to enable technology developers to have a deeper understanding of sometimes large and diverse target audiences. These user archetypes are developed through quantitative and qualitative user experience research and are particularly useful communication tools for developers to grasp the needs of marginalized or hard-to-reach target users [11-13]. As such, personas, especially ones that leverage biopsychosocial data from multi- or mixed-method inquiry, are an appropriate yet largely underutilized component of current mHealth development [13]. For health services, the value of developing personas lie in their potential to allow for better design of future user-centric mHealth interventions.

This study is the development phase of a proposed mHealth intervention, the txt2heart trial, to support patient adherence to medications for atherosclerotic cardiovascular disease (ASCVD). This is an area of much-needed support as, globally, many individuals with risk factors for ASCVD remain undiagnosed, untreated, and uncontrolled [14-16]. Although medication has shown to be effective in preventing cardiovascular events associated with ASCVD, patients may exhibit suboptimal adherence due to the long-term management required, disease misperceptions, poor health literacy, poor patient-physician relationship, weak social support, and comorbidities [17-21]. The txt2heart trial is an international collaboration evaluating the efficacy and safety of SMS text messaging on clinical outcomes and adherence in different countries, including Colombia, Ghana, India, and Singapore. This paper focuses on work conducted in Singapore where, based on the high mobile penetration locally and available data, we may be able to employ personas to gain a greater understanding of the preferences and behaviors of the target population.

Our multi-method behavioral study sought to explore a variety of participant experiences with ASCVD and associated risk factors such as hypertension. Our paper aims to put forth personas that illustrate the variety of both adherence and technology behaviors in Singapore and to propose ways forward for the use of personas in the design and implementation of mHealth interventions.

## Methods

### Sampling and Recruitment

The study took place in Singapore and was comprised of an exploratory quantitative survey and qualitative study using in-depth interviews. For both the qualitative and quantitative component, we used purposive sampling from an existing community health study patient list. This list comprised participants from two neighborhoods in Singapore who participated in a community health study aiming to identify health needs of residents. Of those who consented to recontact, we recruited via telephone those aged 40 years and above with ASCVD or associated risk factors; Textbox 1 lists the complete inclusion and exclusion criteria. Once recruited, surveys and interviews were conducted in person at places of convenience to each participant. There was no overlap between recruitment for the qualitative and quantitative studies.

**Study inclusion and exclusion criteria.**

Inclusion criteria:

1. Patients with a history of atherosclerotic cardiovascular disease (ASCVD): coronary artery disease, ischemic stroke, peripheral artery disease, and atherosclerotic aortic disease OR
2. Patients with at least one risk factor such as hypertension or hyperlipidemia, where antiplatelet, antihypertensives, and/or statins are recommended.

Exclusion criteria:

1. Participants unable to participate in a verbal interview.
2. Participants who did not speak Mandarin, English, or Malay languages.

### Data Collection

Research team members administered the survey instrument and conducted semistructured, in-depth interviews in the participant’s preferred language by staff fluent in that language. Both the survey instrument and interview guide were developed as part of the larger txt2heart collaboration, which we then adapted to the Singapore context (see Multimedia Appendix 1). Both covered topics such as patients’ sociodemographic characteristics, health care access, medication adherence, mobile phone technology usage (ie, ownership, access, and utilization), and interest in mHealth. We have included a consolidated criteria for reporting qualitative research (COREQ) checklist in Multimedia Appendix 2.

The quantitative exploratory component involved 100 participants who took part in either a face-to-face or phone survey as per their convenience and availability. The qualitative component involved face-to-face, semistructured, in-depth interviews with 20 participants. Out of the 20 participants interviewed, 19 agreed to audio recordings and 1 participant declined. For the latter, detailed field notes and an extensive memo were taken for inclusion in data analysis.

### Ethical Considerations

Informed consent for participation and recording was obtained before both the quantitative surveys and qualitative interviews using a participant consent form and information sheet. Participants could refuse to answer any of the questions and/or discontinue participation in the research at any time. Ethical approval was obtained from the Institutional Review Board at the National University of Singapore.

### Analysis

For the quantitative component, all data were analyzed using SPSS Statistics for Windows, version 24.0 (IBM Corp). Frequencies (n) and percentages (%) were used to summarize sociodemographic characteristics, the pattern of mobile phone technology usage, and interest in mHealth.

For the qualitative component, interviews were recorded and transcribed in full. Two research team members coded interviews using NVivo 11 software (QSR International), applying inductive approaches, thematic analysis, and techniques from the constant comparative method, where line-by-line analysis of early interviews is used on subsequent interviews to test preliminary assumptions [22,23]. Reviewers agreed on identified codes and themes, namely barriers and facilitators to adherence, mobile phone usage, attitudes and preferences, health information-seeking behaviors, and willingness to learn. Through these discussions, reviewers agreed that thematic saturation had been reached.

### Persona Development

Using emergent themes from the analysis of the qualitative study, we developed a proforma to guide persona development (see Multimedia Appendix 3). The proforma captures participant demographics, socioeconomic factors, social supports, treatment adherence, health literacy and information seeking, and mobile phone usage; using standardized definitions (see Multimedia Appendix 3), each qualitative interview was analyzed against the proforma to bring forth the salient attributes of each persona.

This proforma enabled us to cluster participants into groups, taking into consideration low-to-high social support, treatment adherence habits, and mobile phone usage, as well as identification of common factors contributing to these. Using both qualitative and quantitative data analysis, we constructed personas that reflect behaviors and characteristics of the user population in Singapore with relevant codes identified by participant ID number to maintain confidentiality and exemplary quotes from the 20 qualitative interviews. While some studies opt to give personas names, faces, and individual characteristics, we have kept our personas as icons and titles in order to maintain the focus on broader trends in user groups rather than construction of individuals.

## Results

### The Population Context

Participant characteristics from the qualitative study are outlined in Table 1. The quantitative component involved a total of 100 interviewer-administered surveys, inclusive of one incomplete survey that had missing data on mobile phone technology usage. Participant characteristics from the quantitative study are outlined in Table 2.

### The Singapore Context

As persona creation must account for larger socioeconomic processes, it is important to take note of key health systems factors in Singapore. Health care in Singapore is generally accessible and affordable, with patients availing themselves of largely subsidized care offered in polyclinics (ie, government-subsidized general practice clinics), by private general practitioners, and even in tertiary care facilities for primary care and management of chronic conditions. Within primary care settings, it is a common practice for doctors to prescribe multiple months’ worth of medication to regular patients; this availability of medication is reported by our participants to facilitate their adherence. Further, the Singapore government subsidizes and offers various accessible schemes for those with chronic conditions; by and large, our participants reported using these schemes to fund their medications and most reported being able to afford their medications.

However, despite these systems-level facilitators to adherence, 65 out of 100 (65.0%) survey respondents reported suboptimal adherence to ASCVD medication as defined by the Medication Adherence Report Scale (MARS-5). Further, our qualitative findings highlighted different patient experiences, including self-titration of medications in response to symptoms and forgetfulness, as playing a role in suboptimal adherence.

### The Technological Context: Mobile Phone Behaviors and Interest

We analyzed our survey data to report mobile phone ownership, access, use, and preferences in our target population, as well as to inform personas that reflect the technological context of a wider population in Singapore.

**Table 1:** Sociodemographic characteristics of participants of the qualitative study (N=20).

Sociodemographic characteristics		Value
Age (years), mean (SD)		72.5 (7.5)
**Age (years), n (%)**		
	61-70	7 (35)
	71-80	8 (40)
	81-90	4 (20)
	Unreported	1 (5)
**Gender, n (%)**		
	Male	12 (60)
	Female	8 (40)
**Ethnicity, n (%)**		
	Chinese	15 (75)
	Indian	4 (20)
	Malay	1 (5)

**Table 2:** Sociodemographic characteristics of participants of the quantitative study (N=100).

Sociodemographic characteristics		Value
Age (years), mean (SD)		65.3 (9.6)
**Age (years), n (%)**		
	<65	45 (45.0)
	≥65	55 (55.0)
**Gender, n (%)**		
	Female	30 (30.0)
	Male	70 (70.0)
**Ethnicity, n (%)**		
	Chinese	66 (66.0)
	Malay	19 (19.0)
	Indian	14 (14.0)
	Other	1 (1.0)

Among 100 surveyed participants, 99 (99.0%) fully completed the survey; 90 out of 99 (91%) participants owned a mobile phone, either an advance-feature or basic-feature mobile phone. Of those mobile phone owners, 70 out of 90 (78%) reported accessing their mobile phones in general at least once a day; the same proportion of patients—70 out of 90 (78%)—reported using SMS text messaging at least once a day. In general, participants predominantly used their phones for phone calls (87/90, 97%), SMS text messaging (60/90, 67%), and other text messaging services, such as WhatsApp (54/90, 60%).

Among mobile phone owners, 48 out of 90 (53%) indicated interest in receiving medication information via mobile phone. Among those interested in receiving medication information via mobile phone, SMS text messaging was the preferred mode of delivery (40/48, 83%).

### Personas

#### Overview

We report our findings as classified into five personas (see Table 3) that have differing awareness of lifestyle factors, such as what constitutes a healthy diet and regular exercise; differing social milieu and social connectedness; a variety of factors contributing to suboptimal treatment adherence; and mobile phone usage, mobile phone usability issues, and willingness to learn about mobile phone features. These factors offer insight into user behaviors in Singapore that may impact user uptake and may be used to inform the design and implementation of mHealth interventions in this population. We have typologized our personas as The Quiet Analog, The Busy Grandparent, The Socializer, The Newly Diagnosed, and The Hard-to-Reach. A visual compilation of persona types can be found in Multimedia Appendix 4.

**Table 3:** Personas overview.

Persona features		Personas				
		The Quiet Analog	The Busy Grandparent	The Socializer	The Newly Diagnosed	The Hard-to-Reach
**Lifestyle factors**						
	Knowledge level of lifestyle factors	Intermediate	Intermediate	High	Low	Intermediate
	Level of social connectedness	Intermediate	High	High	Unclear	Low
**Suboptimal adherence factors**						
	Level of adherence	Suboptimal	Suboptimal	Suboptimal	N/A^a^	Suboptimal
	Suboptimal adherence behaviors	Side effects and polypharmacy	Forgetfulness	Self-titration	Learning to take medications after an acute event	Afraid of acute event
	Example quotes	“I am not taking it...as long as my readings are fine...you know why? Sometimes it gives you aches in your bones. It starts to hurt.” [ID011]	“I tend to forget my medications in the morning on weekends. I take care of my grandchildren; I go to where my daughter stays and so I forget.” [ID004]	“I’m very health conscious. I know how to monitor and adjust...not like those uneducated people who don’t know.” [IDI003]	“I was taking about 11 different medications...At the beginning, there was 14, when I was in the hospital.” [ID007]	“Sometimes I don’t take one. I'm so frightened, you know. I can take two or what, I don’t know but I then forget thinking I got take or what.” [ID014]
**Mobile phone use and needs**						
	Level of mobile phone usage	Low	Intermediate	High	High	Low
	Mobile phone needs	Phone calls only	Calls and SMS^b^	Calls, SMS, WhatsApp, and video	Calls, SMS, WhatsApp, and video	Only for calls when out
	Level of interest in using a mobile phone	Low	Intermediate	High	High	Low
	Level of interest in mHealth	Low	Low	Intermediate	High	Intermediate
	Desires	Wants to maintain own routine	Wants information from a trusted source	Wants tailored content	Wants to learn how to manage	Wants information for peace of mind
	Example quotes	“There is a lot of stuff in the mobile phone but it’s very difficult for us to learn how to use them. It’s not easy.” [ID001]	“Maybe when they [government] send, I am more comfortable.” [ID016]	“It would help if it’s in Chinese, Malay, Tamil. Those of us who are older don’t understand English and thus the content in the SMS.” [ID009]	“It [SMS reminder] will be useful for patients who just came out of the ward.” [ID007]	“I find myself having the habit of worrying while taking care of myself. Thus, I don’t listen much to the words of others.” [ID005]

^a^N/A: not applicable.

^b^SMS: short message service.

#### Persona A: The Quiet Analog

The first persona explores a patient who largely shies away from uptake of technology and has low interest in an mHealth intervention (see Table 4). Based on the 20 qualitative interviews, 6 (30%) of our participants exhibited low mobile use and a preference for face-to-face contact with health care providers or traditional media for consumption of health information. Participants also reported physical barriers to using their phones such as poor eyesight, which may make it difficult to read SMS text messages. While these patients exhibited suboptimal adherence for a variety of reasons, they did not see mHealth as a solution to these issues; indeed, one participant indicated that medication consumption was a choice dictated entirely by the importance one places on that medication. As these patients exhibit lower mobile phone usage and are not actively seeking health information, they may require a longer time to familiarize themselves with an mHealth intervention and may benefit from being introduced to mHealth by a health care provider.

**Table 4:** Persona A: The Quiet Analog.

Persona features		Details
**Lifestyle**		
	Features	May have some mobility issues but attempts to have a good diet and get exercise Independent in managing their conditions
	Example quote	“I always go [to the clinic] by myself. No need to make trouble for others.” [ID001]
**Social supports**		
	Family support	High
	Friend support	Unclear
**Adherence factors**		
	Barriers	Forgetfulness Side effects Polypharmacy Self-titration
	Facilitators	Medicine supply Cost
	Example quote	“But if I go out, I won’t bring and eat it. It’s very troublesome to take it when I’m on the bus or MRT^a^. I’ll just miss taking it.” [ID018]
**Health information seeking**		
	Level	Intermediate
	Actions	Gets information during consultations with general practitioners and from pharmacists Reads about health topics in the newspaper Does not seek out health information
	Example quote	“They can be seen on the newspaper, but you cannot believe in all of them. I think the most important thing is that they are not trustworthy. You need to think about whether what they say is correct and true.” [ID001]
**Mobile usage**		
	Level	Low
	Example quote	“I have a mobile phone but don’t really use it. My children gave it to me. I didn’t put the phone card into it.” [ID001]
**Usability concerns**		
	Concerns	Low interest in using phone Physical barriers
	Example quote	“Our eyesight is also failing. Sometimes we see an ‘8’ as an ‘S.’ It also makes it difficult to read our SMS^b^.” [ID014]
**Attitudes toward mHealth**		
	Attitudes	No interest in mHealth Happy with current phone usage
	Example quote	“No. As if they want to take medication, they will. Some people, they’re not taking it on purpose. I don’t take some of my medication too. We take only the important medicine and don’t take those that we feel aren’t important.” [ID018]

^a^MRT: mass rapid transit.

^b^SMS: short message service.

#### Persona B: The Busy Grandparent

The second persona explores a patient who uses their mobile phone regularly and expresses interest in an mHealth intervention but wants information from a trusted source (see Table 5). A total of 4 of our participants out of 20 (20%) exhibited or largely exhibited intermediate mobile phone use and a diverse range of health information sources, including online sources. While this persona may use their mobile phone, they may not be interested in using it for mHealth, as their phones may be largely for direct communication with their children. Other usability barriers included receiving spam, which may cause them to inadvertently filter out mHealth SMS text messages. This also speaks to their need to trust the information source, as they will only pay attention to known messengers. These patients reported suboptimal adherence, often due to busy schedules from taking care of grandchildren. As these patients are already on their mobile phones, it is important to leverage the ways they currently interact with SMS text messaging. Participants reported liking the SMS text message reminder services from the polyclinic, so while they may report that they do not think an mHealth intervention is useful, in practice they may find similar utility in such a service as they do with their current SMS text message reminders.

#### Persona C: The Socializer

The third persona explores a patient who uses their mobile phone regularly and they may, themselves, disseminate health information to their diverse social circles (see Table 6). A total of 5 of our participants out of 20 (25%) exhibited intermediate or high phone use and described their large family and social networks. This persona is physically and socially active and strives to lead a healthy life. This persona uses their phone to stay connected, browse the Internet, and regularly interacts with a host of traditional and digital information sources; thus, they may prefer content that is tailored to their needs. One usability concern is the need for services in languages other than English.

**Table 5:** Persona B: Busy Grandparent.

Persona features		Details
Lifestyle features		Active and advocates for good diet and exercise Mostly independent in managing their conditions
**Social supports**		
	Family support	High
	Friend support	Intermediate
**Adherence factors**		
	Barriers	Busy with other activities Forgetfulness Fear of side effects from long-term use
	Facilitators	Medicine supply Cost Habit and self-organization Proximity to polyclinic
	Example quote	“I tend to forget my medications in the morning on weekends. I take care of my grandchildren; I go to where my daughter stays and so I forget.” [ID004]
**Health information seeking**		
	Level	Intermediate
	Actions	Gets information from various health providers as well as family and friends Reads up online about conditions and confident in coping with chronic conditions
	Example quote	“I’ve been sick for so long, I’m used to taking medications once in the morning, once in the evening, etc. I place the medication on the table every night so that I see it in the morning.” [ID002]
**Mobile usage**		
	Level	Intermediate
	Actions	Uses phone to stay in touch with family and friends, primarily through phone calls and SMS^a^
**Usability concerns**		
	Concerns	Receives a lot of spam messages Low interest in using phone for mHealth Physical barriers
	Example quote	“Firstly, the numbers are very small, my fingers are very thick, so two numbers would be dialed at the same time. Second, I didn’t buy my own mobile phone, my children bought it for me.” [ID002]
**Attitudes toward mHealth**		
	Attitudes	Interested in mHealth SMS text messages from polyclinic but does not think the intervention is useful
	Example quote	“I think whatever method you want to use, it's nothing compared to convincing that patient that it is in their own interest and it is important for them to take the medicine regularly according to the schedule. Right?” [ID019]

^a^SMS: short message service.

**Table 6:** Persona C: The Socializer.

Persona features		Details
**Lifestyle**		
	Features	Active lifestyle, both physically and socially Independent in managing their conditions
	Example quote	“People don’t need to tell me. I know myself.” [ID006]
**Social supports**		
	Family support	High
	Friend support	High
**Adherence factors**		
	Barriers	Busy with other activities Side effects
	Facilitators	Habit and self-organization Medicine supply Cost Proximity to polyclinic Instructions written in preferred language
	Example quote	“The pharmacist told me how to take them so I can manage them myself. Since the medication is meant for yourself, then you need to manage it yourself as well.” [ID009]
**Health information seeking**		
	Level	High
	Actions	Seeks information from multiple sources (eg, health care providers, media, the Internet, friends, and family) and often disseminates that information to their family and various social circles
	Example quote	“Cause why you know when they see my SMS^a^, I said, ‘Oh, she said, don't take this.’ Then after they see me, they say, ‘Good you remind me of visit, my cough lessen.’” [ID012]
**Mobile usage**		
	Level	High
	Actions	Uses phone to stay in touch with family and friends through calls and WhatsApp Uses various apps
**Usability concerns**		
	Concerns	Language concerns
	Example quote	“It would help if it’s in Chinese, Malay, Tamil. Those of us who are older don’t understand English and thus the content in the SMS.” [ID009]
**Attitudes toward mHealth**		
	Attitudes	Interest in mHealth
	Example quote	“This is the, what is it, the IT world. Everything must learn.” [ID012]

^a^SMS: short message service.

These patients have a high interest in mHealth, some already even use health apps, and would require an intervention that does not simply tell them what to do, as they already feel a high degree of agency in self-management.

#### Persona D: The Newly Diagnosed

The fourth persona explores a patient who has been newly diagnosed with a chronic condition (see Table 7). While only 1 participant out of 20 (5%) described the challenges of the learning curve in medication use, it is an important stage to consider for an mHealth intervention for treatment adherence. The majority of our participants attributed their ability to adhere to habit formation and a self-determined system for organizing and coordinating their medication. The time after diagnosis is a crucial time for the development of these habits and one that may require more intense support. This persona is coming to terms with a new diagnosis and, after being discharged, is managing multiple medications on their own for the first time. This persona may need reassurance and support to enable habit formation and to understand any side effects; thus, an mHealth intervention may meet their needs and allow continuity of care from inpatient to outpatient settings.

**Table 7:** Persona D: The Newly Diagnosed.

Persona features		Details
Lifestyle features		May have low knowledge of lifestyle factors or an inactive lifestyle Unsure of their condition and may be dependent on others to help manage
**Social supports**		
	Family support	Unclear
	Friend support	Unclear
**Adherence factors**		
	Barriers	Transition from inpatient to outpatient care Polypharmacy Creating new routines
	Facilitators	Fear of acute event or recurrence of acute event Contact with health care professionals Medicine supply Cost
	Example quote	“I was taking about 11 different medications...At the beginning there was 14, when I was in the hospital.” [ID007]
**Health information seeking**		
	Level	High
	Actions	Learning how to manage Receiving information from multiple sources
**Mobile usage**		
	Level	High
	Actions	Uses phone regularly
Usability concerns		No specific concerns
**Attitudes toward mHealth**		
	Attitudes	High interest in mHealth to help learn how to manage
	Example quote	“It [SMS^a^ text message reminder] will be useful for patients who just came out of the ward.” [ID007]

^a^SMS: short message service.

#### Persona E: The Hard-to-Reach

The final persona explores a patient who is disconnected, both technologically as well as socially (see Table 8). This persona may have limited support to lead an active lifestyle and make healthy diet choices; while they are independent in managing their condition, it may not be by choice, due to low family and social supports. A total of 4 of our 20 participants (20%) exhibited characteristics that typify this persona. This persona may be motivated by fear; this fear may indeed facilitate adherence as a way to prevent acute events, but it may also cause them to worry and, thus, skip doses if they forget if they have taken their medication. While they may seek information from health care providers, these interactions are often sporadic, and they may lack other social touch points for health information. This persona may have low literacy, both technological as well as reading literacy, and may have challenges learning to use their mobile phones, as they may not have the resources to teach themselves how to use SMS text messaging or other apps. Some participants in this category reported using and liking their current appointment SMS text message reminder services; it may be beneficial to use a similar format and content, as well as to explain an mHealth intervention for adherence or information in the health care setting from a trusted source.

**Table 8:** Persona E: The Hard-to-Reach.

Persona features		Details
**Lifestyle**		
	Features	Limited support to lead active lifestyle Independent in managing their conditions but not always by choice
	Example quote	“But I have no choice. Who should I call? My niece or nephews? They need to work. It’s better that I go on my own.” [ID005]
**Social supports**		
	Family support	Low
	Friend support	Low
**Adherence factors**		
	Barriers	Side effects Polypharmacy
	Facilitators	Habit and self-organization Medicine supply Cost Motivated by fear
	Example quotes	“Sometimes I don’t take one. I'm so frightened, you know. I can take two or what, I don’t know but I then forget thinking I got take or what.” [ID014] “Because I’m scared to die, it motivates me to eat my medication, without even any reminder...I used to take care of old people, my grandmother, and I saw them suffering.” [ID008]
**Health information seeking**		
	Level	Intermediate
	Actions	Seeks information from health care providers Has low connectedness to other sources beyond the media May have lost or not have access to important sources of health information and reminders
	Example quote	“Every time she gives me call and I remember [to take]; she suddenly pass away, one month now. That’s why I think, nobody helps me.” [ID013]
**Mobile usage**		
	Level	Low
	Actions	Calls only May read SMS^a^ text message but cannot reply
**Usability concerns**		
	Concerns	Low literacy: cannot read the characters and limited English Challenges to learning
	Example quote	“I’ve heard that we need to chase up with technology. But do you think everyone went to school? If you teach me what this is, I’ll forget what that was.” [ID005]
**Attitudes toward mHealth**		
	Attitudes	Interest in mHealth, but only for SMS appointment reminders
	Example quote	“Every time I cannot see the [appointment] card...so the [text] message comes, it’s easy for me.” [ID014]

^a^SMS: short message service.

### Persona Application

These five personas present insight into our target user group for a treatment adherence intervention in Singapore. Based on these personas, we can see variability in multiple dimensions, including lifestyle and social factors, adherence factors, and mobile technology use and needs. These will be useful across the project process and will help to better refine the intervention implementation and dissemination strategy [11,24].

For example, within our population, The Socializer persona may provide an accessible group to initially focus on and engage with, as members of this group seek and disseminate health information via mobile phone. As this group has high social connectedness, they may also act as *influencers* and provide a platform for scale-up and buy-in from their social networks over time. Thus, initial message content design may be tailored to and tested with this group and may account for their belief in their abilities to self-manage and their perceived high health literacy.

Conversely, both The Busy Grandparent and The Hard-to-Reach personas more keenly seek information from familiar trusted sources as compared to The Socializer; thus, when considering how to onboard users, these groups may prefer to be introduced to the intervention through a trusted health care provider. This information helps clarify stakeholders to be involved in the dissemination and diffusion of the intervention.

From an implementation perspective, our personas show that users are not only using SMS text messaging, but also other messaging platforms such as WhatsApp. The feasibility of using such apps for an intervention may be worth exploring in the Singapore context. We also see how The Quiet Analog persona prefers to use their phone for calls only, thus the intervention may require a multiplatform approach in order to reach the widest cross-section of users.

Finally, The Newly Diagnosed persona points toward the utility of the intervention in a previously unconsidered group; this provides the opportunity to explore a modified or targeted intervention to onboard patients at the point of diagnosis or shortly thereafter. While the different personas provide insight into the different ways that users engage with mobile phone use, they should be used as a guide rather than a direct prescription of how an intervention should change, given that the context in which these personas operate do influence an intervention’s efficacy.

## Discussion

### Principal Findings

Our work explores the creation of biopsychosocial personas and how these may highlight different patterns of use and inform the design of mHealth interventions for adherence, as they assist in communicating the diversity in behaviors, preferences, and needs that can be represented through their use. Further, our study explores these personas in the unique context of Singapore.

Singapore has a rapidly aging population and increasing prevalence of chronic disease, which require innovative approaches to disease management [25]. With a high rate of mobile phone usage and digital penetration, as demonstrated by the 91% mobile phone ownership in our sample, Singapore is an ideal candidate for mHealth interventions. Therefore, our research provides a handle for technology designers to tackle behavioral variability in adherence among older adults.

Adherence behaviors are inherently complex and dependent on multiple factors, including the health system, socioeconomic factors, the medication itself, patient factors, as well as factors related to the condition being treated [26]. These factors were evident in our study sample, particularly with regard to health system factors, including ease of access and affordability of medications. Of note is the challenge in promoting adherence for asymptomatic chronic conditions such as hypertension and hyperlipidemia; indeed, our participants demonstrated self-titration and suboptimal adherence to these medications. Other studies have found similar findings, pointing to the challenge of promoting adherence to these medications and the need for innovative patient supports for adherence [27].

While mHealth shows promise in providing a far-reaching and cost-effective approach to facilitate adherence [28-30], caution is warranted in implementing these interventions indiscriminately to target populations. For example, in our study, participants reported a wide range of mobile phone usage behaviors, and even those who had high mobile use did not necessarily want to receive an mHealth intervention for their ASCVD medications. Patient-centered care seeks to provide appropriate services that address the unique needs and preferences of individuals; as such, mHealth approaches should adopt the user-centered design tenet of “know thy user” and use best practices in design to create appropriate and effective interventions in line with user needs and preferences [31]. For example, the majority of participants, when asked in the interview, reported not wanting an mHealth intervention for adherence; however, many reported liking an SMS text message appointment reminder. Thus, there is the potential to gain buy-in from these users as they are already using a similar service.

There is no one solution for digital approaches and users must be understood and contextualized. This is important when attempting to bridge the digital divide and provide mHealth interventions to aging populations who are not “digital natives” [32-34]. For example, our personas highlight the multiple usability issues reported by this population, including wanting content in languages other than English and physical barriers to use such as poor eyesight. We also highlight users who use their phones in specific ways, such as only for phone calls to family or for a wide range of activities such as games and videos, but who feel empowered to manage their own medications. To this end, health researchers must strive to consider a diversity of older adult user behaviors, including user motivations, attitudes, and preferences, in order to design effective and appropriate interventions for adherence to ASCVD medications.

Personas allow a middle path to highlighting the diversity of the user base in a manageable and actionable way. While it may not be feasible to design and implement mHealth interventions for the preferences or adherence patterns of each individual, as evidence has shown, it is also unwise to design for a user group that has been reduced to a homogenous typology, such as *adherent/nonadherent* or *elderly* [35]. Personas can then be used to begin discussions on appropriateness during the intervention design phase, enabling the creation of more robust user journeys by prompting discourse on how a given persona may react to a certain task, prompt, or call to action, and by providing reminders of who is being designed for throughout the research process [24,36]. As noted in our personas, a few groups expressed low interest in using their phone to assist in their medication taking. We include these personas as it is important to consider why certain groups may not want to take up an mHealth intervention and to move toward either strategies to gain their buy-in or different interventions to support them.

As the use of mHealth becomes increasingly popular, health researchers will need to adopt *design thinking* approaches and research methods, such as persona creation, in order to better provide contextually appropriate health interventions [13,24]. For example, a study by LeRouge et al explored the creation and use of personas in the Chinese Aged Diabetic Assistant app and showed how devoting time to the creation of personas in the early stages of an mHealth or eHealth (electronic health) intervention can help to shape design requirements, influence functional requirements, and guide the choice of appropriate channels for implementation and diffusion resulting in a contextually appropriate and acceptable intervention [24]. Thus, personas are an important bridging step to facilitate design discussions and allow important data on user experiences and perception to inform and shape the design process of mHealth interventions. Indeed, user-centered design aligns with the ethos of people-centered care; therefore, methods that better enable program designers and implementers to frame programs with diverse user groups in mind are an important piece to creating meaningful and effective mHealth services to promote adherence [37].

### Strengths and Limitations

A strength of our study is the multidisciplinary project team and multi-method study design, which provides greater exploration of nuanced personal experiences and perspectives impacting adherence and preferences. Further, our study includes participants from multiple ethnic backgrounds, giving perspectives in both English, Mandarin, and Chinese dialects. Finally, to our knowledge, this is the first study of its kind in Singapore to construct personas to inform the design of an mHealth intervention for adherence among ASCVD patients. Further, the results of this study may be of use to inform designers and implementers of mHealth interventions in other Asian urban centers with high mobile phone use.

A limitation is that our study excluded individuals with disabilities that prevented them from participating in verbal interviews. Thus, we are missing the views of persons who are hard of hearing or with other disabilities who may benefit from an mHealth intervention for adherence. More research is needed in designing mHealth interventions and creating personas that reflect the unique needs of individuals living with disabilities. Also, our research focused primarily on mobile phone use for the purpose of an SMS text messaging intervention; with increasing mobile phone penetration, it would be worthwhile to explore in greater depth the use and feasibility of other messaging services (eg, WhatsApp and Line) for mHealth interventions. Further, as The Newly Diagnosed persona exemplifies, there is a need to explore interventions for those who are newly diagnosed to support them in achieving optimal adherence.

### Implications for Future Research and Development

Future research in this area should include greater exploration of user journeys among diverse elderly groups to better understand how and when is best to deliver mHealth interventions. These studies should leverage additional user experience research techniques, including user acceptance and usability testing, as well as traditional public health study designs, such as longitudinal studies with routine follow-up, to determine the impact of messaging to different groups. Unlike persona use in other fields such as marketing [38], there is currently a dearth of robust health research on outcomes and evaluations of interventions that use personas. There is also a need for more multi- and mixed-method, user-centered research that synthesizes evidence on user behaviors and preferences for mHealth interventions for adherence among older persons. Finally, there is a need for greater collaboration between health researchers and those working in digital and creative fields to develop robust and feasible interventions that leverage innovative techniques and best practices.

### Conclusions

As public health and medicine move toward wider adoption of mHealth, it is increasingly important that we pay due consideration to user-centered design techniques, and *design thinking* in order to create meaningful, patient-centered interventions for adherence to medications, as well as to evaluative if these methods result in more effective interventions. Our study leverages multi-method data to explore context, user behaviors, and preferences, and to put forth personas that typify a range of user group experiences to inform the design and creation of mHealth interventions to facilitate adherence to ASCVD medication.

## Appendix

Multimedia Appendix 1 Interview guide.

Multimedia Appendix 2 Consolidated criteria for reporting qualitative research (COREQ) checklist.

Multimedia Appendix 3 Proforma legend and example.

Multimedia Appendix 4 Overview of personas.

## Abbreviations

ASCVD: atherosclerotic cardiovascular disease
COREQ: consolidated criteria for reporting qualitative research
eHealth: electronic health
MARS-5: Medication Adherence Report Scale
mHealth: mobile health
MRT: mass rapid transit
NUHS: National University Health System
SMS: short message service
SPHERiC: Singapore Population Health Improvement Centre
