# Supplementary material for: User Preferences and Persona Design for an mHealth Intervention to Support Adherence to Cardiovascular Disease Medication in Singapore: A Multi-Method Study
Source: JMIR Mhealth Uhealth. 2019 May 28;7(5):e10465. doi: 10.2196/10465 (PMC6658252; doi:10.2196/10465)
Supplement: Multimedia Appendix 1 [file mhealth_v7i5e10465_app1.docx]

| **Introductory Questions:**   - Take demographic information - To start, could you tell us a bit about your family? - Can you tell me a bit about your living situation? Do you live with anyone? Who? - Are you currently employed? - Does anyone help you with your daily tasks like chores or cooking? |
| --- |
| **Medical History and perception of condition**   - Could you tell me about your health history (or medical conditions you have)? [*Follow their narrative to explore the patients’ journey*] - Can you tell me about how you were diagnosed with Cardiovascular/heart disease? - Could you share with me the changes you had to make in daily life after you were diagnosed with CVD/heart disease? - What do you think contributed to your CVD/heart disease? - What did you know about CVD/heart disease before you were diagnosed? |
| **Perception and taking medication**   - Could you share with us all the medicine you currently take? *[Ask if it is possible for us to see the medication, and ask about when and how often they take it]* - What instructions were you given on how to take the medication - Do you feel like taking the medication helps to relief of your condition? - Do you take your medication regularly? - Do you experience any problems in taking the medication? - Do you ever forget to take your medicine? - Does anyone or anything remind you to take your medication? |
| **Health System Perception**   - Moving on, can you tell me about where you go to see the doctor for your CVD/heart disease? - How often do you see your doctor? - Overall, what is your relationship with your doctor like? - Do you talk to any other health care workers for your CVD/heart disease? Nurses? Counsellors? Can you tell me about your relationship with them? - Do you face any difficulties accessing health services? - How do you pay for your medical expenses? - Do you have any problems affording for your CVD/heart disease medication? |
| **Support and information networks**   - Where do you seek information about how to manage your CVD/heart disease? - What kind of information? - Do you use this information frequently? - Do you feel like you have enough information to help you manage your CVD/heart disease? - Does anyone help you manage your CVD/Heart Disease? |
| **Use and appropriation of technology**   - Do you own a mobile phone? - How often do you use your mobile phone? - What do you usually use your mobile phone for? - Do you experience any difficulties using your mobile phone? - Would you find it useful to receive information about different aspects of your treatment through your mobile phone? - Would you find it useful to receive reminders to take your medication through your mobile phone? - How often would you like to receive such reminders? - Would you like the possibility of being able to reply to these messages, where healthcare professionals can respond to your queries? |
