# Supplementary figures and images for: User Preferences and Persona Design for an mHealth Intervention to Support Adherence to Cardiovascular Disease Medication in Singapore: A Multi-Method Study
Source: JMIR Mhealth Uhealth. 2019 May 28;7(5):e10465. doi: 10.2196/10465 (PMC6658252; doi:10.2196/10465)

**
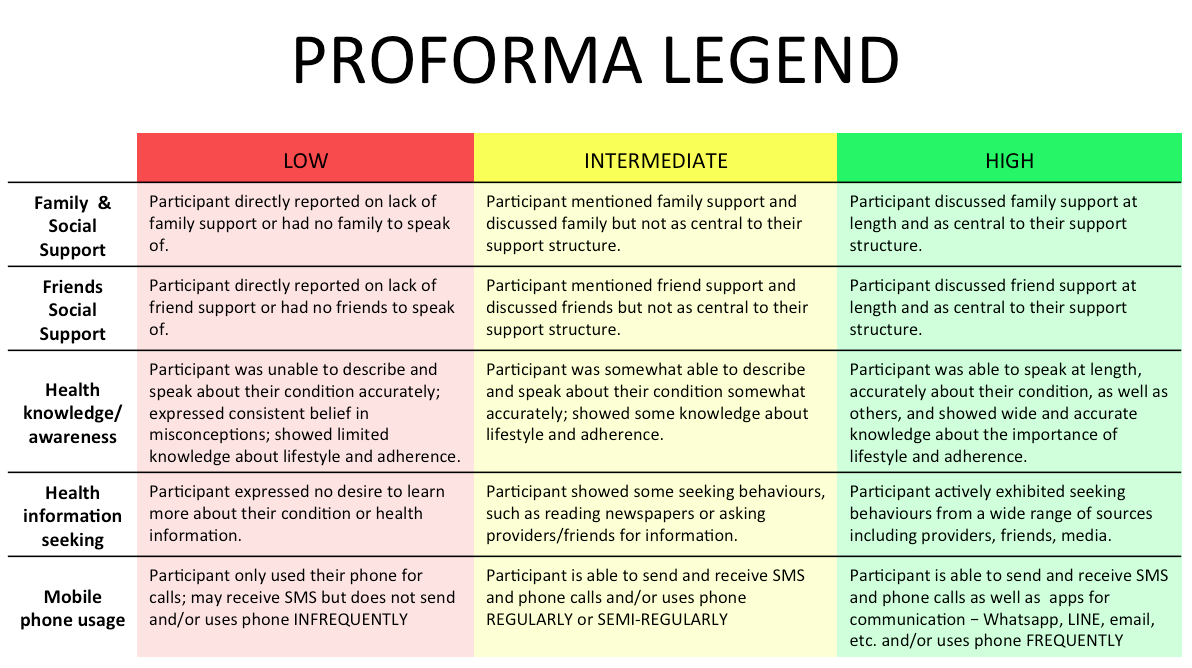

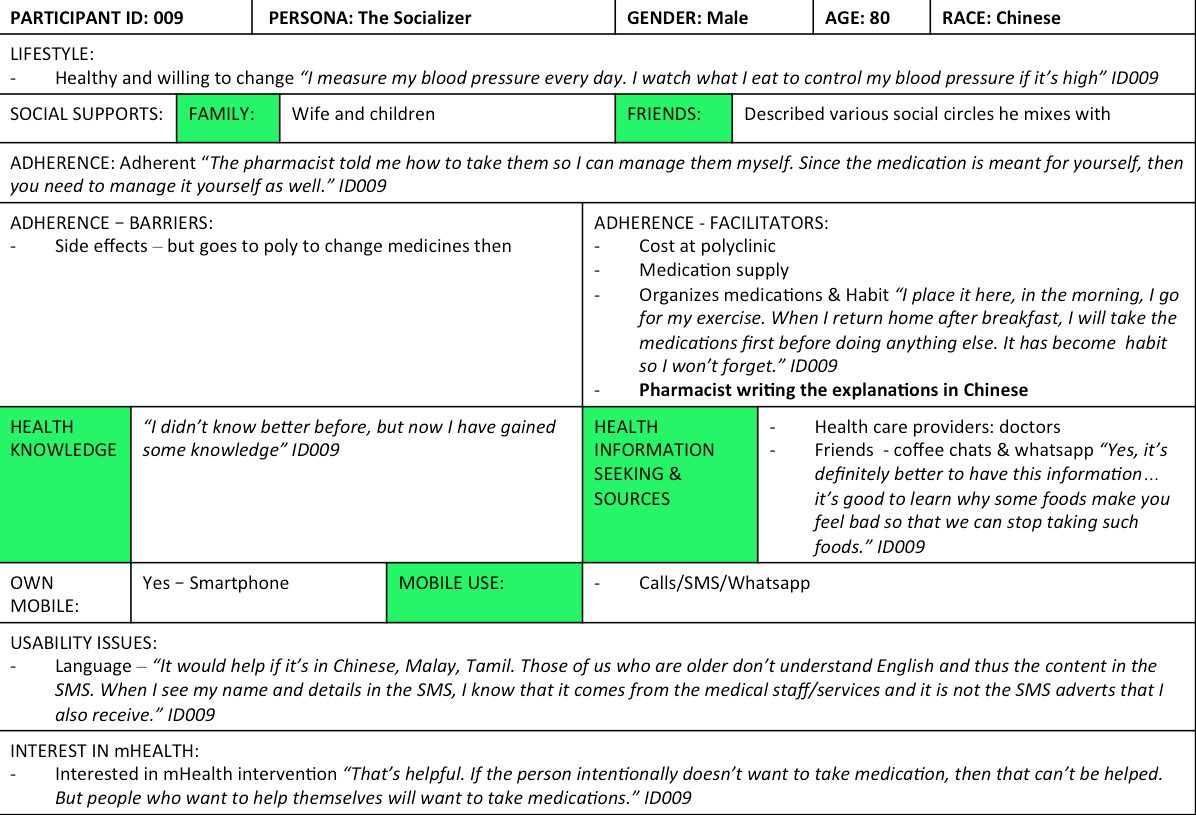
**

Supplement: Multimedia Appendix 3 [file mhealth_v7i5e10465_app3.docx]
