# Supplementary material for: User Preferences and Persona Design for an mHealth Intervention to Support Adherence to Cardiovascular Disease Medication in Singapore: A Multi-Method Study
Source: JMIR Mhealth Uhealth. 2019 May 28;7(5):e10465. doi: 10.2196/10465 (PMC6658252; doi:10.2196/10465)
Supplement: Multimedia Appendix 4 [file mhealth_v7i5e10465_app4.pdf]

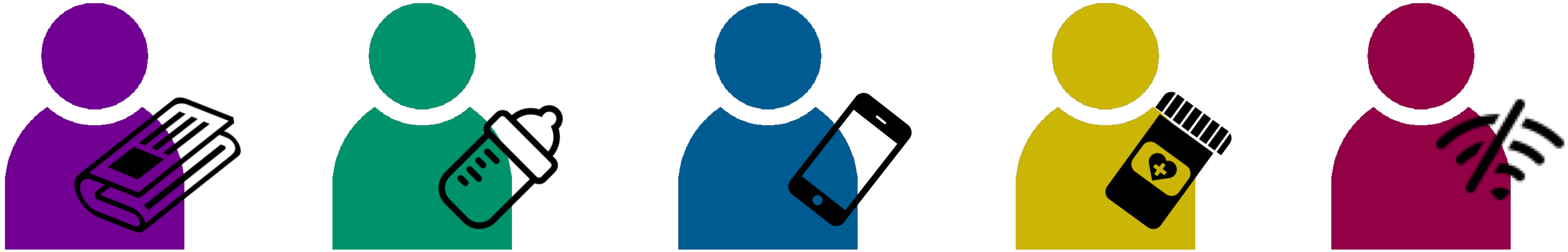

	The Quiet Analog	The Busy Grandparent	The Socializer	The Newly Diagnosed	The Hard to Reach
**Lifestyle & Social**	Intermediate knowledge of lifestyle factors.	Intermediate knowledge of lifestyle factors.	High knowledge of lifestyle factors.	Low knowledge of lifestyle factors.	Intermediate knowledge of lifestyle factors.
	Intermediate social connectedness.	High social connectedness.	High social connectedness.	Unclear social connectedness.	Low social connectedness.
**Sub-optimal Adherence Factors**	Suboptimal adherence - Side Effects & Polypharmacy.	Suboptimal adherence - Forgetfulness.	Suboptimal adherence – Self-Titration.	Learning to take medications after an acute event.	Suboptimal adherence– Afraid of acute event.
	- *“I am not taking it…as long as my readings are fine…you know why? Sometimes it gives you aches in your bones. It starts to hurt.” ID011*	*“I tend to forget my medications in the morning on weekends. I take care of my grandchildren, I go to where my daughter stays and so I forget.” ID004*	*“I’m very health conscious. I know how to monitor and adjust…not like those uneducated people who don’t know.” IDI003*	*“I was taking about 11 different medications…At the beginning there was 14, when I was in the hospital.” ID007*	- *“Sometimes I don’t take one. I'm so frightened, you know. I can take two or what, I don’t know but I then forget thinking I got take or what.” ID014*
**Mobile Use & Needs**	Low mobile phone usage – phone calls only.	Intermediate mobile phone usage – calls and SMS	High mobile phone usage – Calls, SMS, WhatsApp, video.	High mobile phone usage – Calls, SMS, WhatsApp, video.	Low – only for calls when out.
	Low interest in using a phone; **wants to maintain own routine**.	Intermediate interest in using a phone; **wants information from a trusted source.**	High interest in using phone and intermediate interest in mHealth; **wants tailored content.**	High interest in using the phone and high interest in mHealth; **wants to learn how to manage.**	Low interest in using a phone and intermediate interest in mHealth; **wants information for peace of mind.**
	“There is a lot of stuff in the mobile phone but it’s very difficult for us to learn how to use them. It’s not easy.” ID001	*““Maybe when they (government) send, I am more comfortable.” ID016*	*“It would help if it’s in Chinese, Malay, Tamil. Those of us who are older don’t understand English and thus the content in the SMS.”ID009*	*“It [SMS reminder] will be useful for patients who just came out of the ward.” ID007*	*“I find myself having the habit of worrying while taking care of myself. Thus I don’t listen much to the words of others.” ID005*

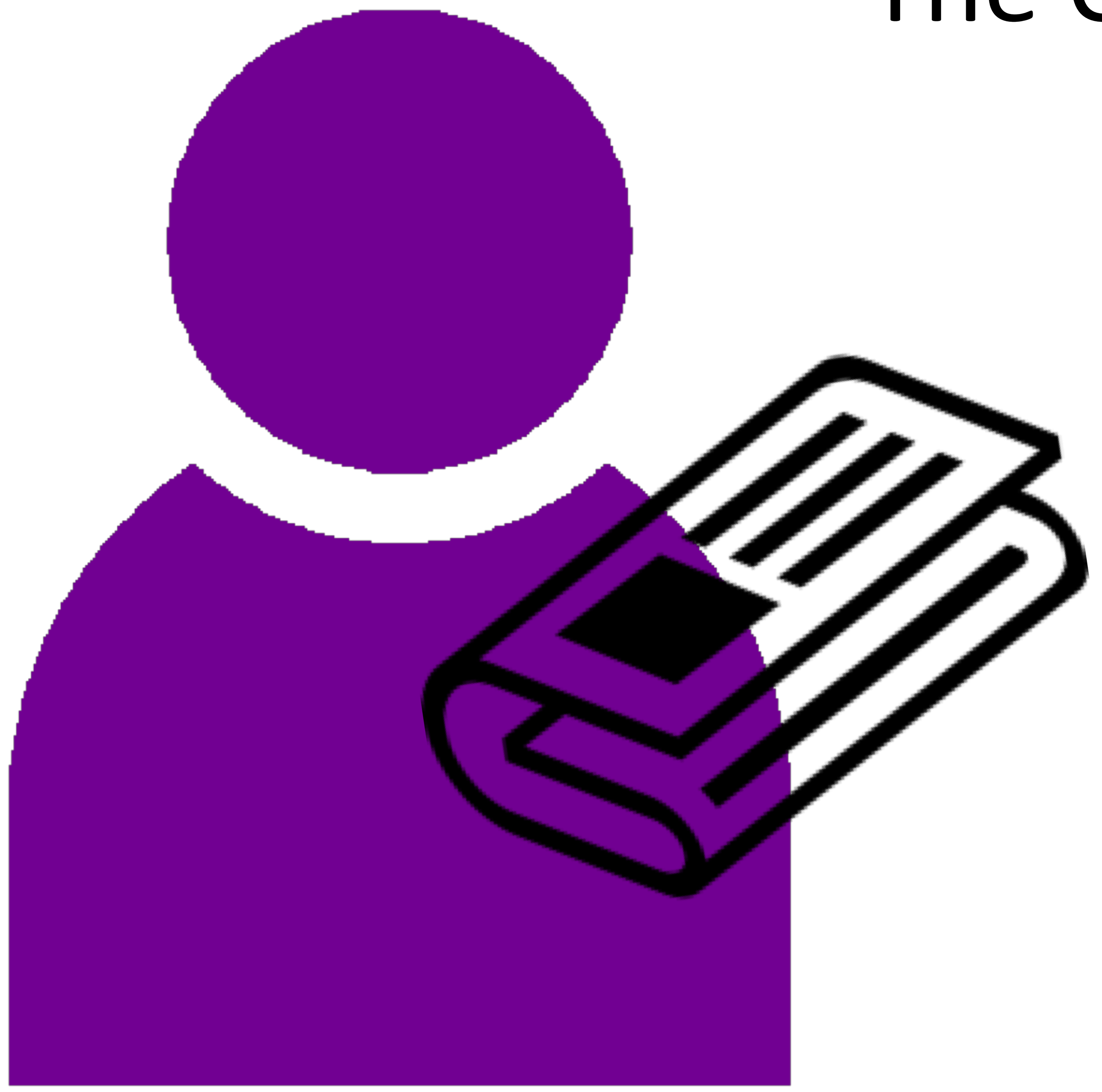

The Quiet Analog

Lifestyle:	- May have some mobility issues but attempts to have a good diet and get exercise<br>- Independent in managing their conditions: *"I always go [clinic] by myself. No need to make trouble for others" ID001*
Social Supports:	Family Support: HIGH<br>Friend Support: Unclear
Adherence Factors:	**Barriers:**<br>• Forgetfulness<br>• Side effects<br>• Polypharmacy<br>• Self-Titration<br>**Facilitators:**<br>• Medicine supply<br>• Cost<br>*"But if I go out, I won't bring and eat it. It's very troublesome to take it when I'm on the bus or MRT. I'll just miss taking it. " ID018*
Health Information Seeking:	INTERMEDIATE: Gets information during consultations with GPs and from pharmacists. Reads about health topics in the newspaper. Does not seek out health information.<br><br>*"They can be seen on the newspaper but you cannot believe in all of them. I think the most important thing is that they are not trustworthy. You need to think about whether what they say is correct and true." ID001*
Mobile usage:	LOW *"I have a mobile phone but don't really use it. My children gave it to me. I didn't put the phone card into it" ID001*
Usability concerns:	Low interest in using phone and physical barriers<br><br>*"Our eyesight is also failing. Sometimes we see an "8" as an "S". It also makes it difficult to read our SMS." ID014*
Attitudes toward mHealth:	No interest in mHealth, happy with current phone usage.<br><br>*"No. As if they want to take medication, they will. Some people they're not taking it on purpose. I don't take some of my medication too. We take only the important medicine and don't take those that we feel aren't important." ID018*

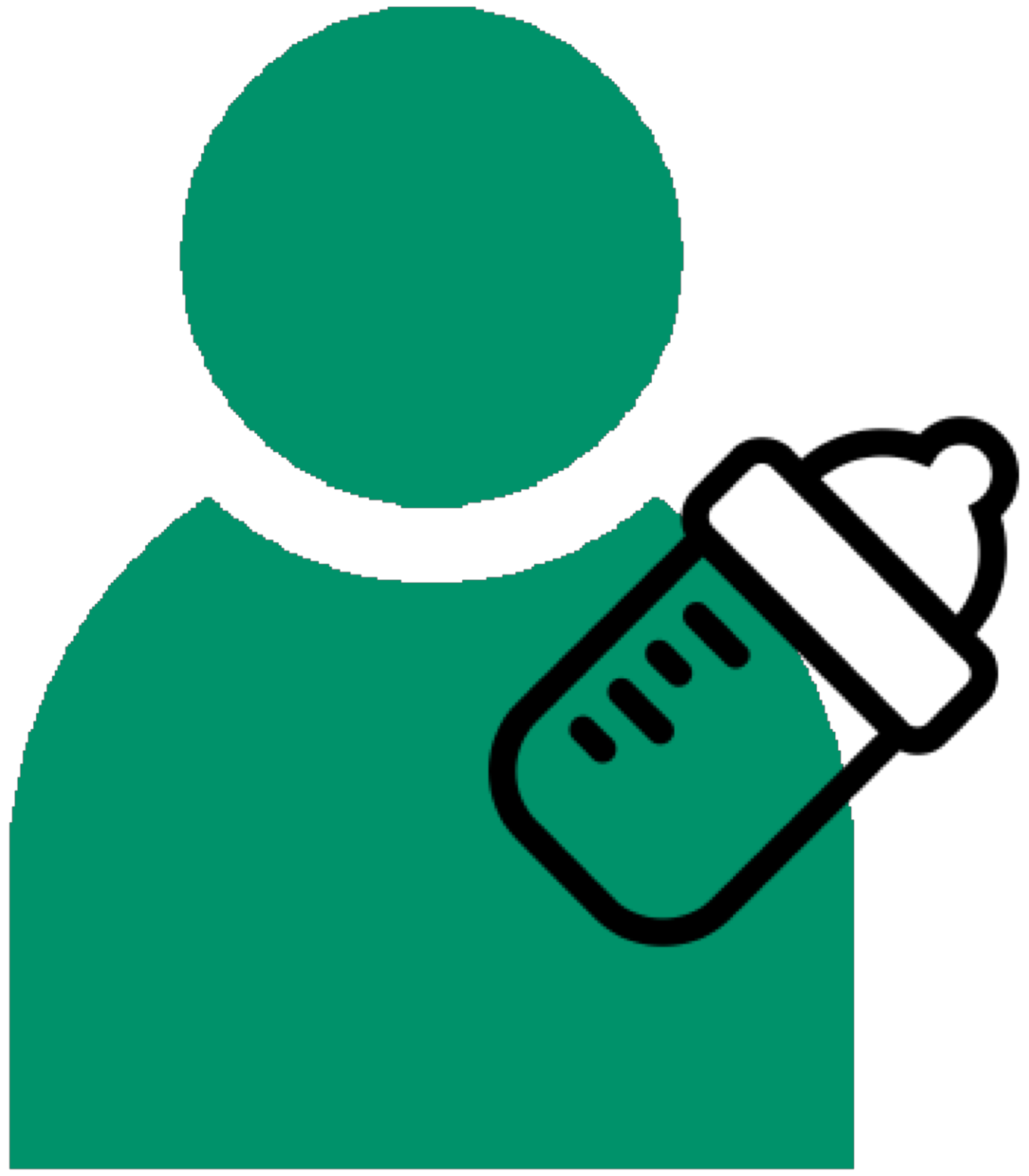

The Busy Grandparent

Lifestyle:
- Active and advocates for good diet and exercise
- Mostly independent in managing their conditions

Social Supports:
Family Support: HIGH
Friend Support: INTERMEDIATE

Adherence Factors:

Barriers	Facilitators
• Busy with other activities<br>• Forgetfulness<br>• Fear of side effects from long term use	• Medicine supply<br>• Cost<br>• Habit and self-organization<br>• Proximity to polyclinic

"I tend to forget my medications in the morning on weekends. I take care of my grandchildren, I go to where my daughter stays and so I forget." ID004

Health Information Seeking:
INTERMEDIATE: Gets information from various health providers as well as family and friends. Reads up online about conditions and confident in coping with chronic conditions.

- *"I've been sick for so long, I'm used to taking medications once in the morning, once in the evening, etc. I place the medication on the table every night so that I see it in the morning." ID002*

Mobile usage:
INTERMEDIATE: Uses phone to stay in touch with family and friends – primarily through phone calls and SMS

Usability concerns:
Receives a lot of spam messages, low interest in using phone for mHealth and physical barriers

"Firstly, the numbers are very small, my fingers are very thick, so 2 numbers would be dialled at the same time. Second, I didn't buy my own mobile phone, my children bought it for me." ID002

Attitudes toward mHealth:
Interested in mHealth SMS from polyclinic but doesn't think the intervention is useful

"I think whatever method you want to use, it's nothing compare to convincing that patient that it is in their own interest and it is important for them to take the medicine regularly according to the schedule. Right?" ID019

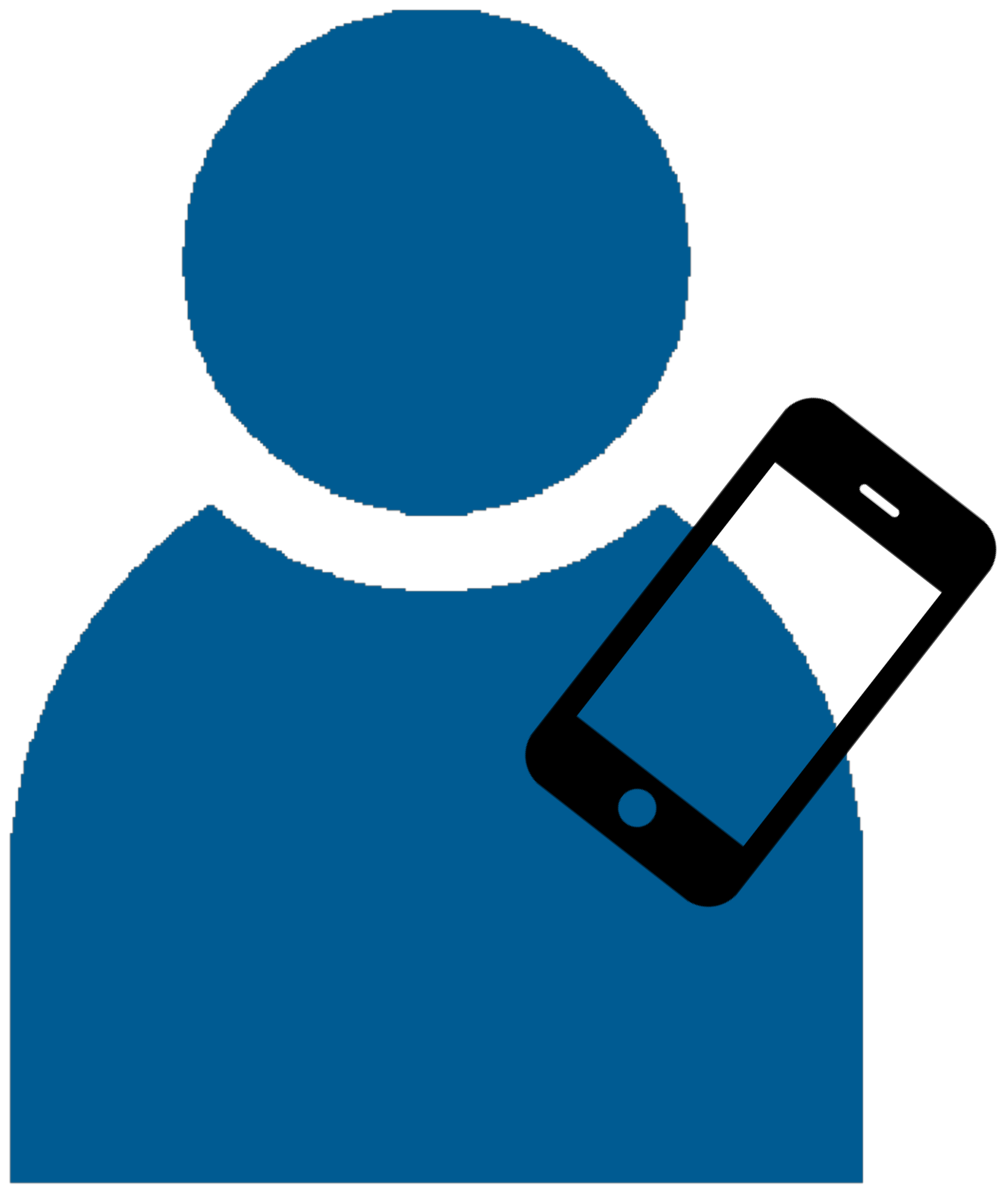

The Socializer

Lifestyle:

- Active lifestyle both physically and socially
- Independent in managing their conditions *“People don’t need to tell me. I know myself.” ID006*

Social Supports:

Family Support: HIGH
Friend Support: HIGH

Adherence Factors:

Barriers	Facilitators
• Busy with other activities<br>• Side effects	• Habit and self-organization<br>• Medicine supply<br>• Cost<br>• Proximity to polyclinic<br>• Instructions written in preferred language

“The pharmacist told me how to take them so I can manage them myself. Since the medication is meant for yourself, then you need to manage it yourself as well.” ID009

Health Information Seeking:

HIGH: Seeks information from multiple sources (health care providers, media, the internet, friends and family) and often disseminates that information to their family and various social circles.

“Cause why you know when they see my SMS, I said, "Oh, she said, don't take this." Then after they see me, they say, "Good you remind me of visit, my cough lessen.”ID012

Mobile usage:

HIGH: Uses phone to stay in touch with family and friends through calls, WhatsApp; uses various apps.

Usability concerns:

Language concerns:

“It would help if it’s in Chinese, Malay, Tamil. Those of us who are older don’t understand English and thus the content in the SMS.” ID009

Attitudes toward mHealth:

Interest in mHealth – *“This is the, what is it, the IT world. Everything must learn.” ID012*

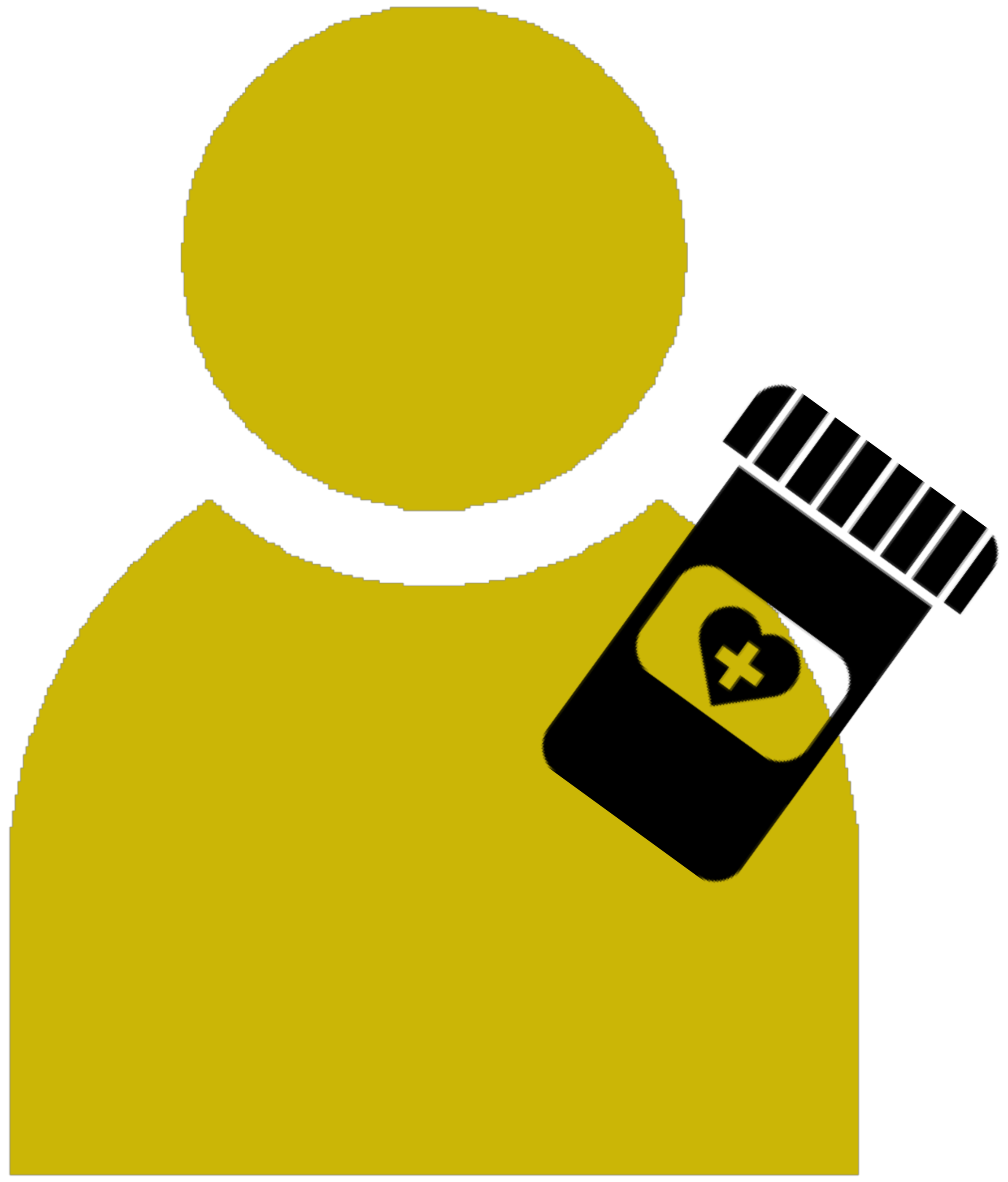

The Newly Diagnosed

Lifestyle:	- May have low knowledge of lifestyle factors or an inactive lifestyle<br>- Unsure of their condition and may be dependent on others to help manage	
Social Supports:	Family Support: Unclear<br>Friend Support: Unclear	
Adherence Factors:	**Barriers**	**Facilitators**
	• Transition from inpatient to outpatient care<br>• Polypharmacy<br>• Creating new routines	• Fear of acute event or recurrence of acute event<br>• Contact with health care professionals<br>• Medicine supply<br>• Cost
	"I was taking about 11 different medications…At the beginning there was 14, when I was in the hospital." ID007	
Health Information Seeking:	HIGH: Learning how to manage and receiving information from multiple sources.	
Mobile usage:	HIGH: Uses phone regularly	
Usability concerns:	None specific	
Attitudes toward mHealth:	High interest in mHealth to help learn how to manage: *"It [SMS reminder] will be useful for patients who just came out of the ward." ID007*	

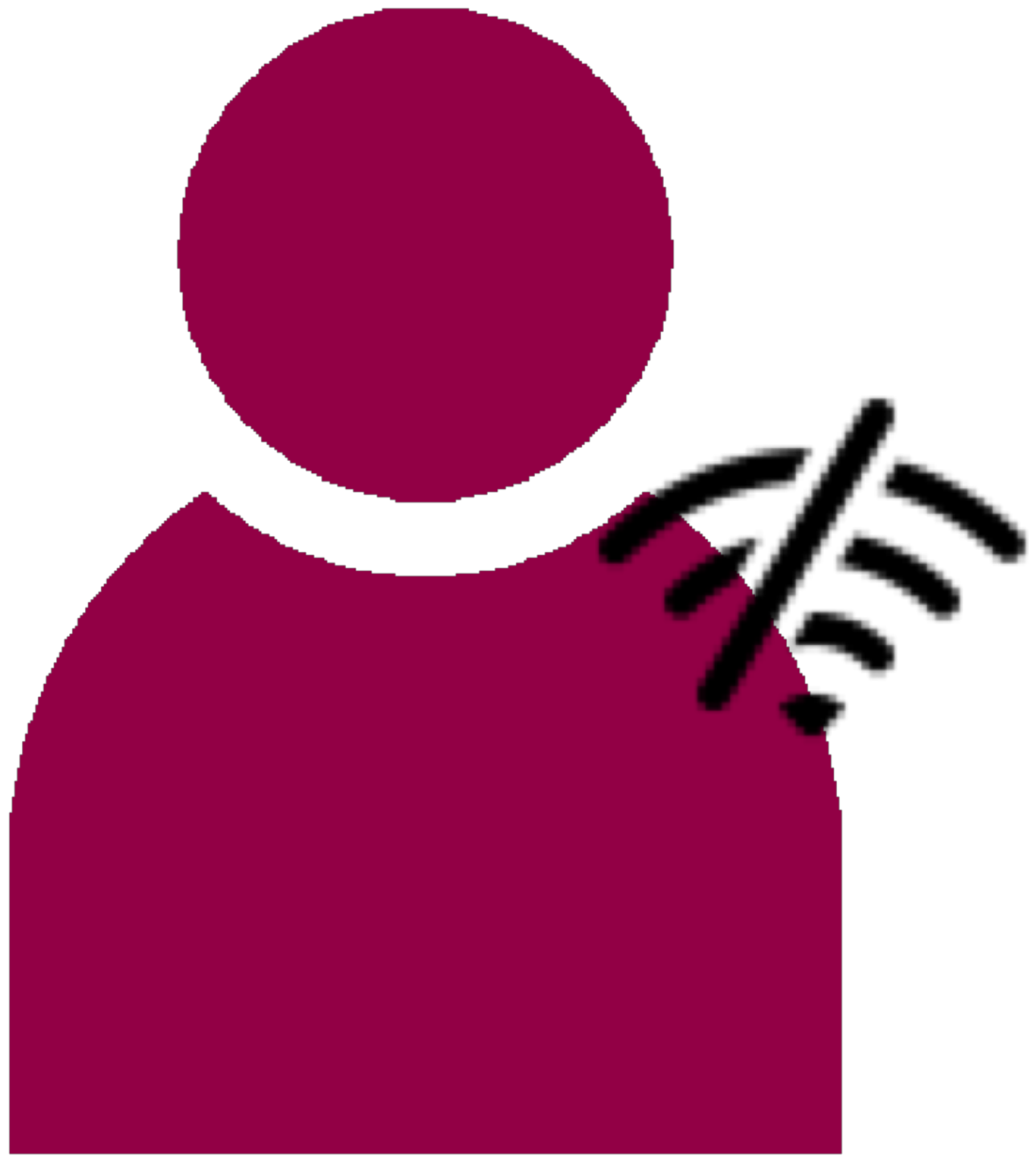

The Hard to Reach

Lifestyle:

- Limited support to lead active lifestyle
- Independent in managing their conditions but not always by choice.

"But I have no choice. Who Should I call? My niece or nephews? They need to work. It's bette that I go on my own." ID005

Social Supports:

Family Support: LOW
Friend Support: LOW

Adherence Factors:

Barriers	Facilitators
• Side effects<br>• Polypharmacy	• Habit and self-organization<br>• Medicine supply<br>• Cost

Motivated by fear in different ways:

*"Sometimes I don't take one. **I'm so frightened, you know. I can take two or what**, I don't know but I then forget thinking I got take or what." ID014*

AND

*"Because I'm scared to die, **it motivates me to eat my medication**, without even any reminder…I used to take care of old people, my grandmother, and I saw them suffering"* ID008

Health Information Seeking:

INTERMEDIATE Seeks information from health care providers, but has low connectedness to other sources beyond the media. May have lost or not have access to important sources of health information and reminders:

"Every time she gives me call and I remember (to take); she suddenly pass away, one month now. That's why I think, nobody helps me." ID013

Mobile usage:

LOW: Calls only, may read SMS but cannot reply

Usability concerns:

- Low literacy – cannot read the characters and limited English
- Challenges to learning *"I've heard that we need to chase up with technology. But do you think everyone went to school? If you teach me what this is, I'll forget what that was." ID005*

Attitudes toward mHealth:

- Interest in mHealth, but for SMS appointment reminders

"Every time I cannot see the (appointment) card…so the ((text) message comes, it's easy for me." ID014
